# The Auto Sensor Test as an AE Signal Source in Concrete Specimens

**DOI:** 10.3390/ma18225084

**Published:** 2025-11-08

**Authors:** Magdalena Bacharz, Michał Teodorczyk, Jarosław Szulc

**Affiliations:** 1 Department of Material Strength and Diagnostics of Structures, Faculty of Civil Engineering and Architecture, Kielce University of Technology, 25-314 Kielce, Poland; 2 Building Structures, Geotechnics and Concrete Department, Instytut Techniki Budowlanej, 00-611 Warsaw, Poland

**Keywords:** acoustic emission, source, AST function, Hsu–Nielsen test, velocity, waveform, wavelet analysis, non-destructive testing, concrete

## Abstract

Numerous artificial sources of acoustic waves have been described in the literature, which are designed to replicate the process by which actual damage occurs in a given material. Knowledge of the velocity with which an acoustic wave propagates is important here, both in order to correctly locate the signal source and to determine the degree of material degradation or the location of damage that has already occurred in the medium. This work presents the results of laboratory tests comparing two sources of artificial waves in terms of determining their parameters: the Hsu–Nielsen source and a sensor with the Auto Sensor Test function. The AST function allows the sensors to send and receive an elastic wave and is used to calibrate the sensor before, during, or after the test. In this study, the impact of the positioning of the sensors on the element being tested, their spacing, and the distance of the wave source from the sensor on selected parameters of the recorded waves are analyzed: velocity, amplitude, energy, rise time, waveform shape, and wavelet maps. This work demonstrates that a sensor with the AST function can be an effective alternative for the Hsu–Nielsen source in diagnostic studies.

## 1. Introduction

### 1.1. Theoretical Background of the Study

Non-destructive diagnostic methods involve assessing the material under study without interfering with its structure and include acoustic methods using elastic wave propagation. Acoustic methods have been applied in the diagnosis of concrete and concrete structures [1,2,3,4]. One of these methods is the acoustic emission method, the idea behind which analyses elastic waves that arise and/or propagate in a structural element in all directions. This method itself does not have a negative impact on the object under study and can be used repeatedly. Research centres around the world are developing measurement methods and techniques using acoustic emission for the diagnosis and analysis of concrete. Examples of research and its results are included, among others, in [5,6,7]. Among the publications, two main research trends can be observed, resulting from the nature of the damage observed, that is, whether cracks [8,9] or microcracks [10,11] were formed during the test, or whether a given crack [12,13] or different destructive processes [14] were formed before the test. This is related to the selection of an appropriate measurement technique. One possibility is to study the active processes that arise in the material as a result of various factors that change the structure of the material, especially those leading to its destruction, for example, the formation of cracks or their propagation. When cracks were formed earlier in the evaluated element (the second discussed case), the test was used to determine the level of material degradation and identify the fracture to take corrective actions. Both approaches are based on an analysis of the elastic wave, its parameters, and its shape.

**Figure 1:**
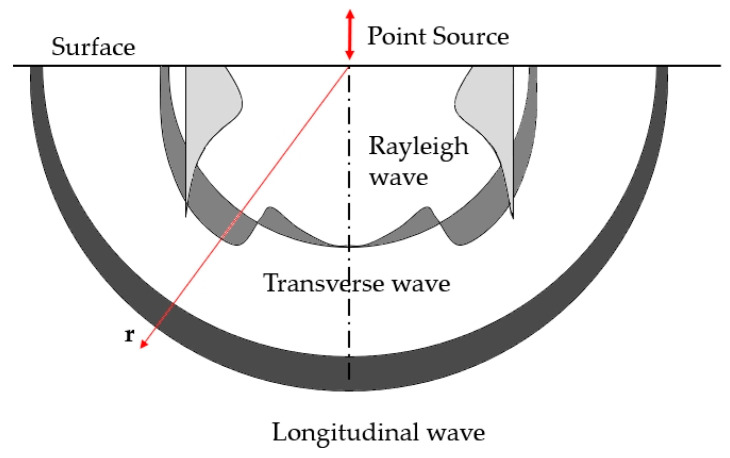
Propagation of three waves as a result of the impact of a harmonic point force on the surface; r—wave propagation path.

The theoretical disturbance of the medium caused by the action of a point force on the surface assumes the formation of three types of waves. These are longitudinal (P-wave), transverse (S-wave), and Rayleigh (R-wave) waves, also known as surface waves [15,16]. Figure 1 shows a diagram of the propagation of these waves in half-space, with their corresponding amplitudes, based on [16,17].

Longitudinal and transverse waves propagate in the medium in a hemispherical form, whereas Rayleigh waves propagate over the surface of the medium [16]. As shown in Figure 1, the longitudinal wave propagates first, followed by the transverse wave, and finally, the Rayleigh wave. Each of these waves is characterised by different velocities and amplitudes of the vibrations. For the longitudinal wave, the distribution of the vibration amplitude is the greatest along the vertical path of radius r (below the point source), whereas for the transverse wave, the distribution of the vibration amplitude is the greatest along the diagonal path of radius r. For Rayleigh (surface) waves, Figure 1 displays the vertical component of the vibration amplitude distribution, which fades as the distance from the surface increases [15,18].

Based on the velocity of the propagation of elastic waves in the material, the acoustic emission method allows the location of the source of active destructive processes. The declared velocity has an impact on the interpretation of the measurement results, for example, the correct location of the damage in an element, whose exact material parameters are often unknown. The value of the wave velocity, for example, in concrete, depends on factors such as the size of the aggregate, occurrence of pores, concrete density, presence of reinforcement, temperature, and humidity. Despite numerous studies from which it is possible to “obtain” the values of the elastic wave velocity in concrete, for example [19], the spread of these values is so wide that the best option is to perform independent measurements.

An artificial point source with impulse characteristics was used to excite an elastic wave to determine its velocity in the material and other parameters [20,21]. The characteristics of the artificial source that induces an elastic wave on the surface of the material should be similar to those of the natural source of acoustic emission. The following are examples of artificial sources with pulsed-type characteristics which produce waves, as shown in Figure 1.

**Figure 2:**
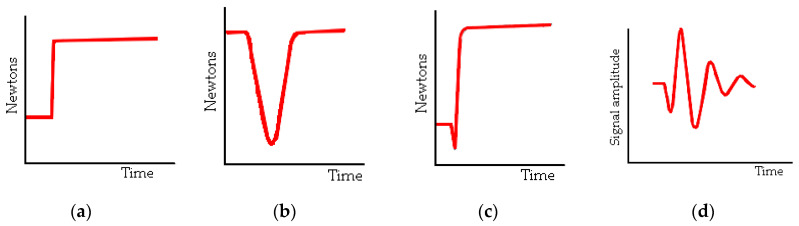
Schematically presented examples of artificial sources of acoustic emission: (**a**) breaking a glass capillary, (**b**) dropping a steel ball, (**c**) graphite cracking, and (**d**) pulse from the sensor.

Examples of artificial sources, schematically presented in Figure 2:

- Breaking the glass capillary
- Dropping a steel ball;
- Hsu–Nielsen test;
- Calibration pulse from an acoustic emission sensor.

The fracture of the glass capillary on the surface is caused by the force described by the step function, Figure 2a. As a result of the pressing force, the glass capillary suddenly broke, generating an elastic wave in the medium. The cross section and mass of the glass capillary were exceedingly small compared to the dimensions and mass of the medium tested [22,23]. The impact of a steel ball on the surface of the medium is caused by the force described by a quadratic function, as shown in Figure 2b. Using a ball with a specific mass and drop height, an elastic wave was excited in the steel plate. It was noted that the greater the mass of the ball, the longer the vibration time of the medium with a constant drop height. Selected studies in this area are described in [20,24]. The Hsu–Nielsen test [25] consists of breaking a graphite lead into a suitable head, causing an elastic wave. The force is described by a step function with a negative peak preceding the wave generation [22,26], as shown in Figure 2c. In addition to receiving the emission signal, some acoustic emission sensors can emit a calibration pulse. This is possible because the measuring apparatus emits a pulse towards the sensor, and the sensor transmits it to the medium. The shape and frequency of the elastic wave depend on the type of sensor selected. The amplitude of the wave vibrations had a sinusoidal shape [22,27], as shown in Figure 2d.

### 1.2. Literature Review and Engineering Significance of the Study

The artificially generated point source allows practical applications in concrete testing to measure wave velocity, analyse the shape of the elastic wave and its parameters, and attenuate the wave amplitude.

Measurement of the velocity of the longitudinal wave is important for determining the properties of the material under study, located the source of acoustic emissions on the surface or in the bulk of the evaluated element. The analysis of the waveform helps identify the arrival time and amplitude of the longitudinal wave and determines the frequency range of the wave. The arrival time of the wave is necessary to measure the wave velocity, and the amplitude of the longitudinal wave is necessary to determine the cause of the acoustic emission source (Moment Tensor analysis [28,29,30]). The attenuation of the wave amplitude is necessary to examine the correct positioning of the sensors or to determine the proper spacing of the sensors when locating the source [27]. The analysis of the recorded signals is possible with the use of the Wavelet Transform (WT) [31]. It is a method based on wavelet analysis that allows signals to be decomposed into components with different frequencies while preserving information about their localisation at a specific time. This allows noise isolation, damage detection, and classification [32], which are especially important in structural diagnostics.

Artificial signal sources are commonly used in the testing of concrete and concrete structures, both through the ultrasonic method [33] and the acoustic-ultrasonic method [34]. Unfortunately, not all artificial sources are characterised by high reproducibility of results. This issue was described in a previous study [35], which pointed out significant standard deviations between the amplitude spectra of signals generated artificially during the pencil test (Hsu–Nielsen test) and when dropping steel balls with diameters of 4 and 5 mm. Therefore, research is being conducted to improve the accuracy of signal source localisation. For example, in [36], the effect of wave velocity attenuation excited by a pencil lead break test was investigated by analysing wave velocity changes depending on the distance between the source and the sensor. A modification of the velocity value based on the attenuation analysis was proposed. In [37], the authors presented the results of research in which they used a 3D localisation algorithm based on the exhaustive method to improve the accuracy of locating the source of the acoustic wave generated in concrete by a pencil test. In [38], it was shown that the use of a pulse generator to excite waves in mortar samples can provide information about changes in the internal structure of the material and may be useful for assessing early damage. In [27], the waveforms recorded during the self-calibration of sensors on bonded composite panel joint samples were analysed, distinguishing between signals before and after loading. It was emphasised that this method should be further developed for use in waveform analysis and damage detection.

The development of research methods supports the assessment of low-emission materials, including modified concrete with additives (e.g., with ceramic dust [39]) and especially geopolymer concrete, as alternatives to high-emission Portland cement. Recent studies have shown that geopolymers can be densified and homogenised, which improves their durability and strength [40] and thus has an impact on the increase in the velocity of the ultrasonic wave [41]. Also taking into account the strong influence of aggregate grading and porosity on the wave velocity in Portland cement concrete [42], it can be assumed that the wave velocity monitoring method (adapted and validated based on the specificity of new materials) can be an effective tool for tracking the hardening process and assessing the quality and condition of geopolymer structures.

### 1.3. Objectives and Scope of the Study

This study aimed to examine the possibility of using the PK6I sensor with a built-in AST (Auto Sensor Test) system to excite and analyse the elastic wave in sample elements and to compare the results with those generated using the Hsu–Nielsen source. The tests were conducted on three cuboid samples with dimensions of 150 mm × 150 mm × 600 mm made of C30/37 grade concrete with basalt aggregate and blast furnace cement. This study analysed the parameters of the elastic wave and its wavelet transform that reached the sensor, considering the method and location of wave excitation and the distance between the source and the receiver. The study compared the results of wavelet analysis and analysis of the parameters of a wave excited in concrete using a sensor with the AST function and employing the Hsu–Nielsen source as the reference. The wave velocity results obtained experimentally were compared with the theoretical values. It was analysed whether the method of acoustic wave excitation by the sensor with the AST function can serve as an alternative to the Hsu–Nielsen method for testing concrete. The novelty of this research lies not only in the methodology (experimental use of the sensors in a way different from that recommended by the manufacturer) but also in its potential to enhance non-destructive assessment of materials, including sustainable construction materials.

## 2. Materials and Methods

### 2.1. Materials

Three cuboid concrete samples with dimensions of 150 × 150 × 600 mm were subjected to tests. The C30/37 grade samples were matured for 10 years in a laboratory room maintained at room temperature. The study of mature concrete, with stabilised parameters, was aimed at verifying the accuracy of the measurement method, its calibration, and establishing a baseline reference level of wave velocity for the prepared mix and aggregate type. The samples were made with basalt aggregate from the Gracze quarry located in Poland at 581 kg (fractions 2–8) and 731 kg (fractions 8–16) per 1 m^3^. The aggregate properties are listed in Table 1.

Moreover, 691 kg of river sand, 180 kg of tap water and 360 kg of blast furnace cement from the Górażdże cement plant located in Poland (whose parameters shown in Table 2) were used in the concrete mixture.

### 2.2. Methods

**Figure 3:**
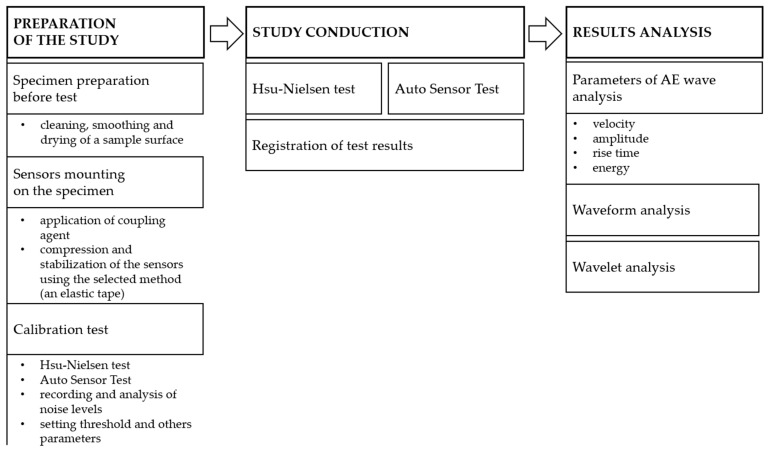
A flowchart of the experimental procedure.

Two methods of acoustic wave excitation were used in this study, which were applied to verify the correct operation of acoustic emission sensors. These include graphite cracking in the Hsu–Nielsen test [46] and the calibration pulse from the PK6I acoustic emission sensor with the auto-sensor test (AST) function. Figure 3 shows a flowchart summarising the experimental procedure.

**Figure 4:**
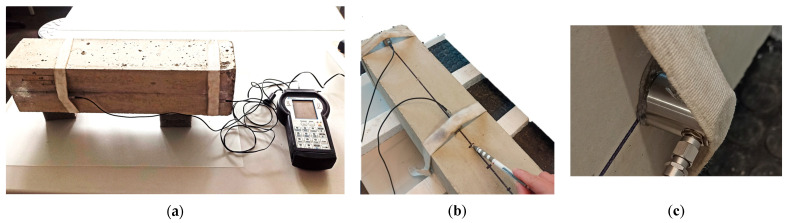
Specimens under test: (**a**) the measurement station; (**b**) the test using graphite (pencil lead) fracture; (**c**) sensor mounted on the sample.

The research was conducted using the Pocket AE^TM^, hand-held acoustic emission system supported by the Vallen Control Panel (R2021.1122.1). Figure 4 shows the measurement station used during the test. The station consisted of two sensors and a portable acoustic emission measuring device. Before commencing the test, the measurement sites on the samples were properly prepared, that is cleaned and smoothed. Contact of the sample with the sensor was ensured by coupling the paste and elastic tape.

The sampling rate of 3 MS/s was used in this study. The sampling rate of 3 MSPS (Mega Samples Per Second) was used in this study. The analysis covered the initial selection of acoustic signals, which was manually performed by the researcher. AE signals were qualified for further analysis based on their characteristic parameters, such as arrival time, amplitude, energy, rise time, and wave shape. The purpose of the selection was to reject “noise” signals so that only signals with a clear onset and unambiguous identification of the wave arrival time could be analysed.

#### 2.2.1. Elastic Wave Excitation Using a Sensor with an AST Function

**Figure 5:**
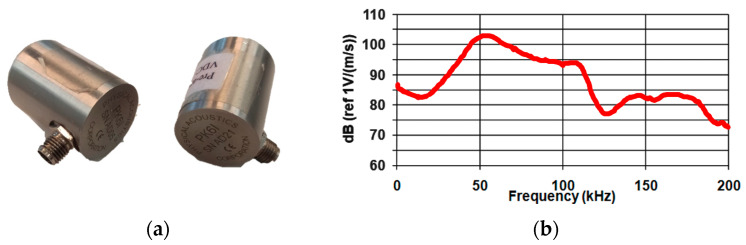
PK6I resonance sensor: (**a**) photograph of the sensors; (**b**) sensor sensitivity graph depending on the frequency.

A PK6I sensor with a resonance frequency of 55 kHz was used in this study because of its practical application in the diagnosis of real concrete structures. The sensor has a built-in 26 dB preamplifier and operates in the 35–65 kHz frequency range. The sensor cooperated with a portable acoustic emission measurement device. The system has an integrated Auto Sensor Test, which allows for emitting an artificial wave of acoustic emission into the medium in the form of sinusoidal tension, as well as receiving waves from the medium at the same time [47]. A photograph of the sensor (Figure 5a) and its frequency response are shown in Figure 5b.

**Figure 6:**
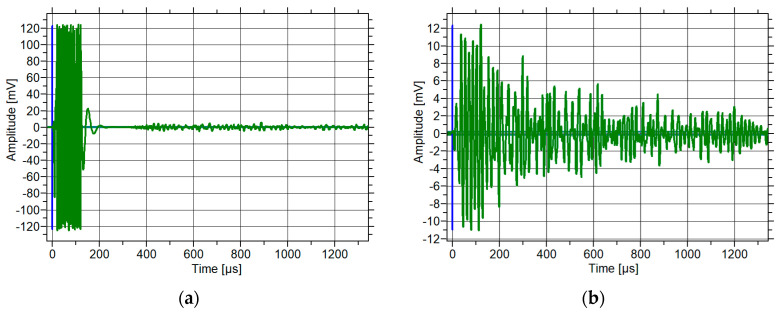
Waveform of elastic wave in concrete sample: (**a**) emitted and (**b**) received by sensor.

The shape of a sample elastic wave recorded in concrete is shown in Figure 6. An elastic wave was emitted by the sensor (Figure 6a) and received by the sensor after propagation in the concrete sample (Figure 6b).

#### 2.2.2. Elastic Wave Excitation Using the Hsu–Nielsen Test

**Figure 7:**
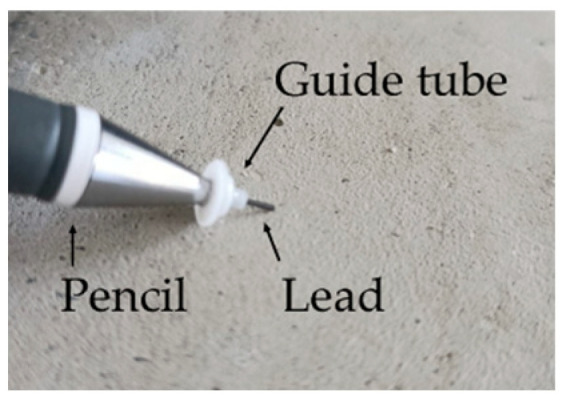
The pencil lead was the Hsu–Nielsen source.

The Hsu–Nielsen source consists of simulating an acoustic emission event as a result of the fracture of a brittle pencil lead held in an appropriate tube, i.e., the protruding lead should be 3.0 ± 0.5 mm long, 0.5 mm in diameter and possess a hardness of 2H (Figure 7). The Hsu–Nielsen source has been standardised in the European standard PN-EN 1330-9 [46] as well as in the American standard [48].

A Teflon ring was fitted onto the mechanical pencil. The ring and protruding lead are inclined at an angle of 30°. When pressure is applied to an obliquely positioned ring, the lead breaks and generates an elastic wave. Therefore, a relatively repeatable reference source can be obtained, as described in [26,49].

**Figure 8:**
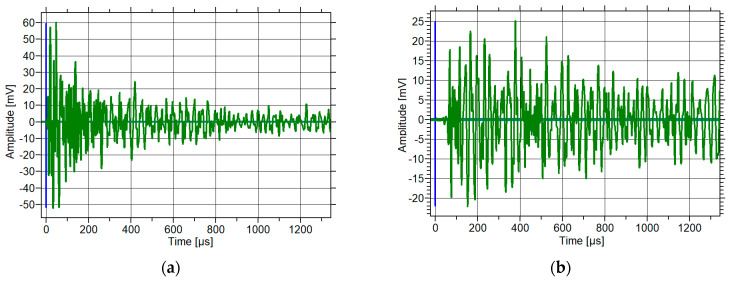
Waveform of elastic wave generated by graphite (pencil lead) fracture: (**a**) at the sensor and (**b**) after its propagation through a concrete sample.

The waveforms of the elastic waves recorded by acoustic emission sensors in concrete are presented below. The Hsu–Nielsen source was excited at the sensor. The resulting waveform (Figure 8a) and the waveform after its propagation through the concrete sample (Figure 8b) are shown below.

### 2.3. Placement of Measurement Sensors

#### 2.3.1. Test Using a Sensor with an AST Function

**Figure 9:**
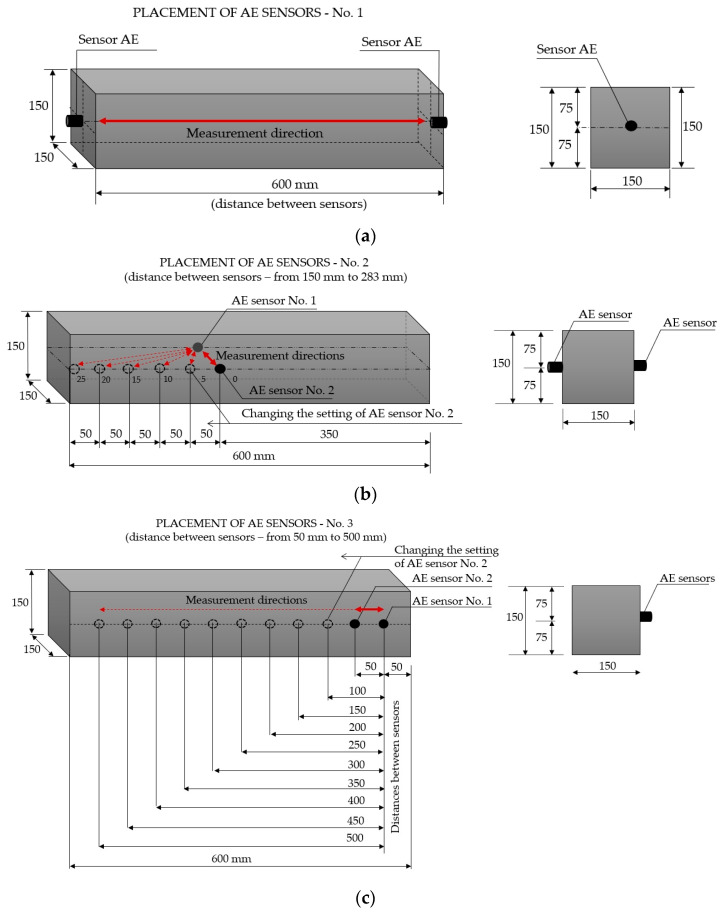
The placement of AE sensors during the test: (**a**) No. 1 direct transmission; (**b**) No. 2 direct transmission; (**c**) No. 3 indirect transmission.

An elastic wave was generated using a sensor with an auto-test sensor function. The positioning of the sensors is shown in Figure 9. Three configurations of sensor positioning were analysed on three concrete samples (III-A, III-B, and III-C) to investigate changes in the velocity of the elastic wave and its parameters (amplitude, rise time, and energy), and the wavelet transform WT, developed as a Gabor wavelet based on the Gaussian function.

In configuration No. 1 (Figure 9a), the sensors were placed opposite each other to obtain a direct measurement of the acoustic wave velocity to analyse its parameters. The sensors were positioned at the centre of gravity on the opposite sides of a given sample at a distance of 600 mm.

In configuration No. 2 (Figure 9b), the sensors were positioned facing each other to obtain a direct measurement but in the opposite direction (at a distance of 150 mm). In addition, the test examined how the sensor displacement may affect the results obtained. For this purpose, during subsequent measurements, the distance between the sensors was increased by moving one of the sensors away from the “0” position, in which the sensors were separated by 150 mm. Hence, subsequent measurements were carried out at the measurement points: “5”, “10”, “15”, “20” and “24”, corresponding to the displacement of the sensor No. 1 over the surface of the sample by 50 mm, 100 mm, 150 mm, 200 mm and 240 mm, respectively. Each displacement resulted in an increased distance between the sensors, respectively: 150 mm (at point “0”), 158.1 mm (at point “5”), 180.3 mm (at point “10”), 212.1 mm (at point “15”), 250 mm (at point “20”) and 283 mm (at point “25”).

In configuration No. 3 (Figure 9c), the sensors were placed on the same side of the concrete sample at half the height of the side to obtain an indirect measurement; that is, the sensors were oriented toward the inside of the sample. To obtain the most accurate estimation of wave velocity, the distance between the sensors was increased in 50 mm increments, starting from 100 mm to 500 mm. The measured lengths and elastic wave velocities were estimated according to the approach described in the standard [50] to determine the ultrasonic wave velocity.

In each sensor configuration, the wave was excited 10 times using the AST function from sensor No. 1 (with sensor No. 2 serving as a receiver) and 10 times from sensor No. 2 (with sensor No. 1 serving as a receiver). The threshold was set at an amplitude of 45 dB.

#### 2.3.2. The Test Using Graphite (Pencil Lead) Fracture (Hsu–Nielsen Source)

**Figure 10:**
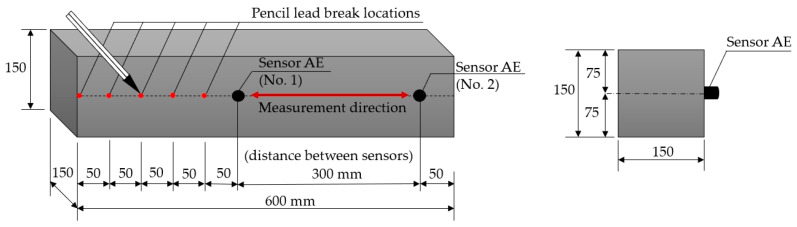
AE sensor placement during the Hsu–Nielsen test.

An elastic wave was excited using the Hsu–Nielsen source in three concrete samples (III-A, III-B, and III-C). During the test, the acoustic emission sensors were placed 300 mm apart on the sidewall of the sample, as shown in Figure 10. The pencil lead fracture was performed at five points at distances of 50, 100, 150, 200, and 250 mm from sensor No. 1, each time repeated at least ten times. Both PK6I sensors recorded the parameters of the received acoustic waves. If any noise occurred when breaking the pencil lead, for instance, due to the pencil lead sliding over the surface of the sample, the corresponding measurement was discarded and repeated to minimise technical inaccuracies.

Changes in elastic wave velocity (generated by the Hsu–Nielsen source) and its parameters (amplitude, rise time, energy), as well as the wavelet transform, were analysed to compare them with the results described in Section 2.3.1.

## 3. Results

### 3.1. Results of AST Function Analysis

#### 3.1.1. Analysis of Acoustic Wave Parameters—Direct Measurement

An acoustic wave velocity test using the AST function was performed in three configurations, as shown in Figure 9. Direct measurements along the sample were obtained based on configuration No. 1 (Figure 9a), while the direct measurement in the opposite direction was obtained based on configuration No. 2 at position “0” (Figure 9b).

**Figure 11:**
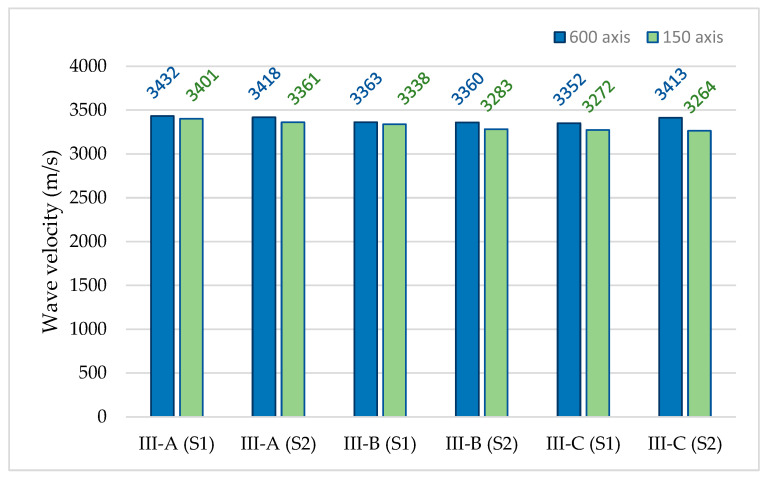
The results of the mean velocity of acoustic waves generated by using the AST function in samples III-A, III-B, and III-C by two sensors placed opposite each other at distances of 600 mm and 150 mm.

Figure 11 shows a comparison of the acoustic wave velocity results recorded in three concrete samples by two sensors placed on each sample at distances of 600 mm (configuration No. 1) and 150 mm (configuration No. 2, measurement “0”). These values were determined from 10 measurements conducted in each configuration with a coefficient of variation below 1%, indicating high repeatability of the results.

In the three concrete samples, similar values of acoustic wave velocity were recorded by all six sensors. The differences between the mean velocity values obtained for each sample in percentage points, along with the statistical parameters calculated for the entire dataset, are presented in Table 3.

The approximate velocity values obtained from direct measurements of concrete in two perpendicular directions (differing below 2.5%) indicate low variability of the results, and therefore, high homogeneity of the material in terms of composition, density, or compaction of the concrete mix, but also no internal defects. In addition, they indicate the repeatability of the measurement method in terms of the test procedure and the operation of the measuring devices (they indicate, among others, the appropriate coupling of sensors with the ground). Similar velocity values indicate that the same type of elastic wave, that is, a longitudinal wave, was recorded.

**Figure 12:**
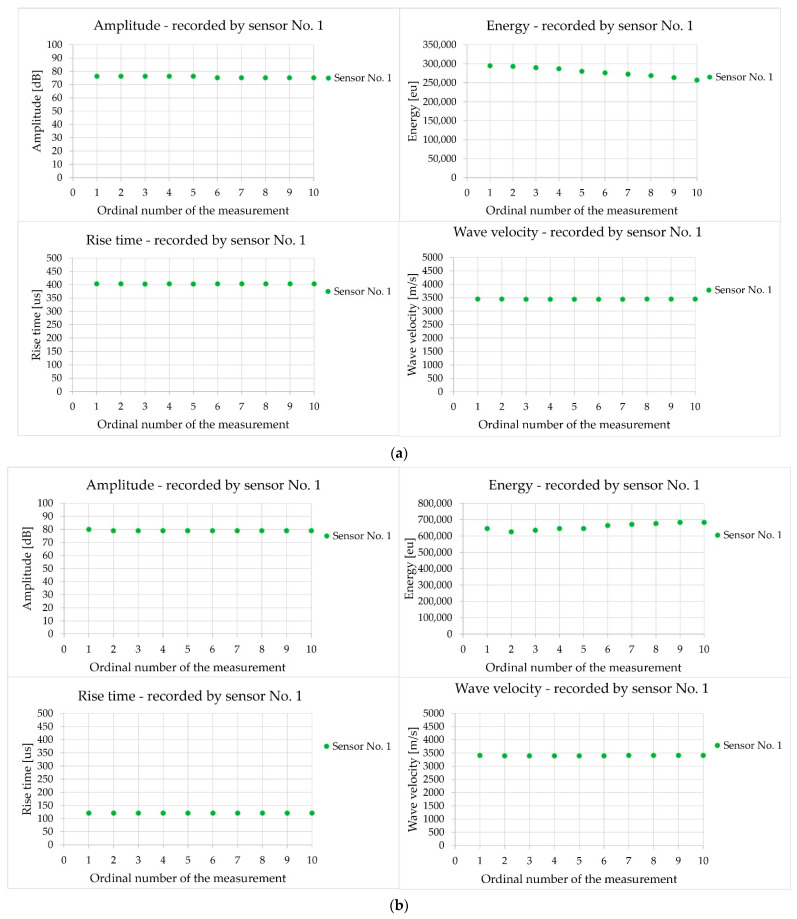
The results of the amplitude, energy, rise time, and velocity of the acoustic wave generated by the AST function during 10 consecutive measurements were recorded by the sample sensor in sample III-A at the axial position of the sensors: (**a**) at a distance of 600 mm; (**b**) at a distance of 150 mm.

Figure 12 shows a summary of the acoustic wave parameters: amplitude, energy, rise time, and velocity of the acoustic wave recorded by the sensor during one measurement (10 consecutive pulses) in demonstration sample III-A (in Figure 12a with the sensors at a distance of 600 mm and in Figure 12b at a distance of 150 mm).

Analysis of the wave parameters demonstrated that the change in the sensor spacing from 600 mm (Figure 12a) to 150 mm (Figure 12b) doubled the wave energy and reduced the wave rise time by more than threefold, with an almost unchanged amplitude and wave velocity. The main reason for these changes is the shorter propagation path of the elastic wave, which reduces the losses. The signal energy increased by 136% as the material attenuation increased with distance, and there was less concrete heterogeneity over a shorter propagation path to dissipate the wave. The rise time decreased by 70% because there were fewer reflections from the edges and fewer overlapping waves over a shorter distance, which lengthened the beginning of the waveform at a distance of 600 mm. The amplitude decreased by 3%. This parameter, expressed in decibels, is less sensitive than the energy, which may be related to the masking of differences by the measuring equipment. In both tests, the velocity of the wave remained unchanged, with a difference of 1%, because it depended on the properties of the material, and the geometry of the measurement path was a simple section that was not affected by adverse phenomena such as reflections. The results obtained using the AST function were characterised by high repeatability. Subsequent measurements performed at the same measurement point were characterised by an extremely low coefficient of variation, below 1% in the case of rise time, amplitude, and wave velocity.

**Figure 13:**
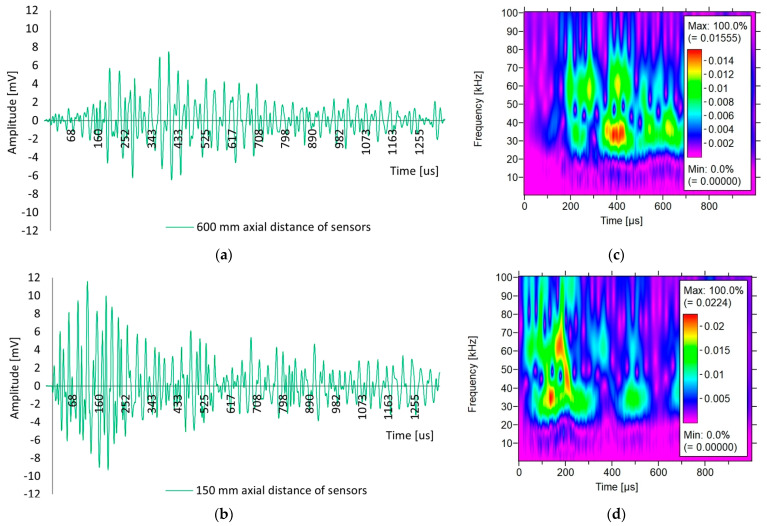
The results of the waveform analysis investigating the acoustic wave generated in sample III-A by the AST function with the sensors positioned in relation to each other: (**a**) axially at a distance of 600 mm, (**b**) axially at a distance of 150 mm, and the results of the wavelet analysis with the WT coefficient scale generated by the AST source of the acoustic wave generated in sample III-A by sensor No. 1 positioned axially in relation to each other; (**c**) at a distance of 600 mm; (**d**) at a distance of 150 mm.

Figure 13 shows the shape of the acoustic wave received by the sensor arranged axially with respect to the other one at distances of 600 mm (Figure 13a) and 150 mm (Figure 13b). Figure 13 also shows the results of the wavelet analysis for the selected sample III-A for an acoustic wave recorded by sensors at distances of 600 mm (Figure 13c) and 150 mm (Figure 13d). Individual images show the results of wavelet transform analysis (WT) on 2D and 3D time-frequency maps received by one of the sensors. The colours on the maps indicate the intensity of the wavelet transform coefficients of the sensor signal.

Based on Figure 13, differences in the shape of the waves received by the sensor can be observed depending on the distance between the sensors. In the case of a longer propagation path, that is, 600 mm (Figure 13a), a rise time of 403 µs and an amplitude of 7 mV were observed, whereas in the case of a distance of 150 mm (Figure 13b), the rise time was 121 µs and the amplitude was 11 mV. The difference in the wave shape resulted from a shorter propagation path. The shorter distance of 150 mm retained high frequencies and reduced attenuation, resulting in a high-amplitude peak with a steep and narrow pulse. A longer distance of 600 mm smoothies and extends the wave at low frequencies, resulting in a smaller amplitude peak and a longer and wider pulse.

Based on Figure 13c,d, differences in the WT values can be observed for the waves received by the sensor, depending on the axial spacing of the sensors. In the case of a longer propagation path, that is, 600 mm (Figure 13c), a low coefficient of 0.015 was observed, whereas for a distance of 150 mm (Figure 13d), the coefficient was higher than 0.022, which resulted in an increase of 46%. Based on the analysis, it appears that a shorter 150 mm wave propagation path causes a higher intensity, the energy is at the beginning of the wave, and the visible ridges reach low frequencies of 25 kHz and Hz, with a dominant frequency of 35 kHz. The longer wave propagation path of 600 mm causes a lower intensity, energy appears later in time, and is distributed over several ridges, reaching lower frequencies of approximately 35 kHz.

**Figure 14:**
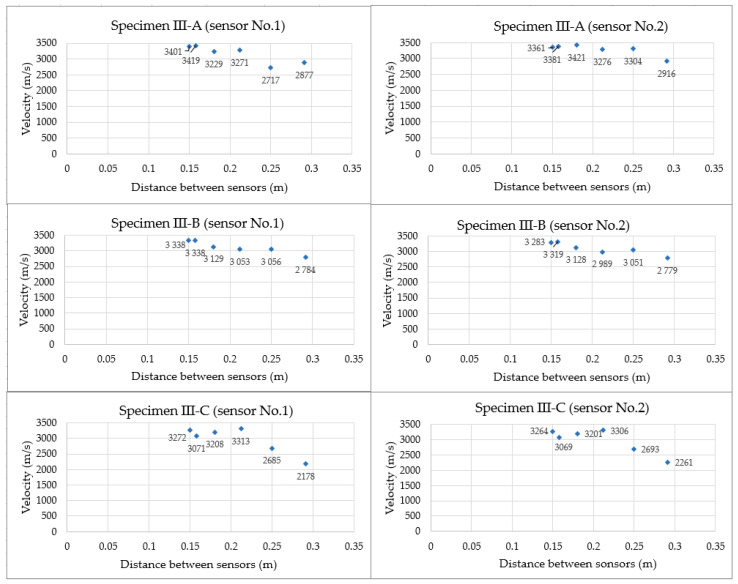
The acoustic wave velocity values were recorded (received) by six AE sensors on three concrete samples depending on the distance between the sensors.

The study also analysed whether the lack of axial positioning of the sensors and their conscious displacement from the direct position affect the values obtained for the velocity of the acoustic wave generated by the AST function. This is important in cases where it is impossible to position the sensors on opposite sides of the element at the points where they directly face each other. After determining the velocity of the acoustic wave recorded by the sensors arranged opposite to each other so that the centre of gravity of the sensors overlapped (setting “0” shown in Figure 9b, at which the distance between the sensors was 150 mm), one of the sensors was moved away from the position “0” five times, according to the diagram shown in Figure 9b, thus increasing the distances between the sensors that were determined using the Pythagorean theorem. The results of the test of the velocity of the acoustic wave received by sensor No. 1 (the wave was then generated by sensor No. 2) and, successively, sensor No. 2 (the wave was generated by sensor No.1), placed on three concrete samples, depending on the distance between these sensors, are shown in Figure 14.

Based on the results shown in Figure 14, it can be concluded that similar acoustic wave velocity values were obtained for all measurements, with a maximum deviation in the sensor axes of up to 100 mm and a distance of 180.3 mm between the sensors. The maximum difference in percentage points between the velocity values measured at position “0” (sensor distance—150 mm) and “10” (sensor distance—180.3 mm) was 4.3%, while between position “0” and “15” (sensor distance 212.1 mm) it was slightly higher at 6.2%. When the sensors were moved away from their axes by 200 mm (sensor distance 250 mm), the wave velocity recorded by both sensors in sample III-C and sensor No.1 in sample III-A clearly decreased to approximately 2700 m/s. When the sensor was moved away by 240 mm (sensor distance 283 mm), a lower acoustic wave velocity was recorded for all samples. Table 4 summarises the calculated mean AE wave velocity obtained for a tilt between the sensor axes of up to 100 mm (distance between the sensors, 180.3 mm) in the evaluated samples, along with the statistical parameters. The coefficient of variation was determined between the mean values calculated from 3 measurements, when the sensors were at positions: “0”, “5” and “10”, i.e., at the positions where similar acoustic wave velocity values were recorded.

In all samples, the coefficient of variation of the velocity was up to 3%, indicating a low dispersion of results around the mean, and demonstrating that small sensor displacements (within a radius of up to 100 mm) from a single-axis arrangement during the test did not significantly affect the obtained acoustic wave velocity values. In the case of larger sensor displacements from their axes (with an initial spacing of 150 mm), one should consider the possibility of obtaining underestimated velocity values, which may result, for example, from attenuation or dispersion in concrete.

#### 3.1.2. Analysis of Acoustic Wave Parameters—Indirect Measurement

To determine the parameters of the wave excited using the AST function on the side wall, sensors were arranged along the length of the sample at distances from 50 to 500 mm in 50 mm increments, according to the diagram shown in Figure 9c. Each measurement was performed ten times, and the coefficient of variation at each measurement point did not exceed 1% for either sensor.

**Figure 15:**
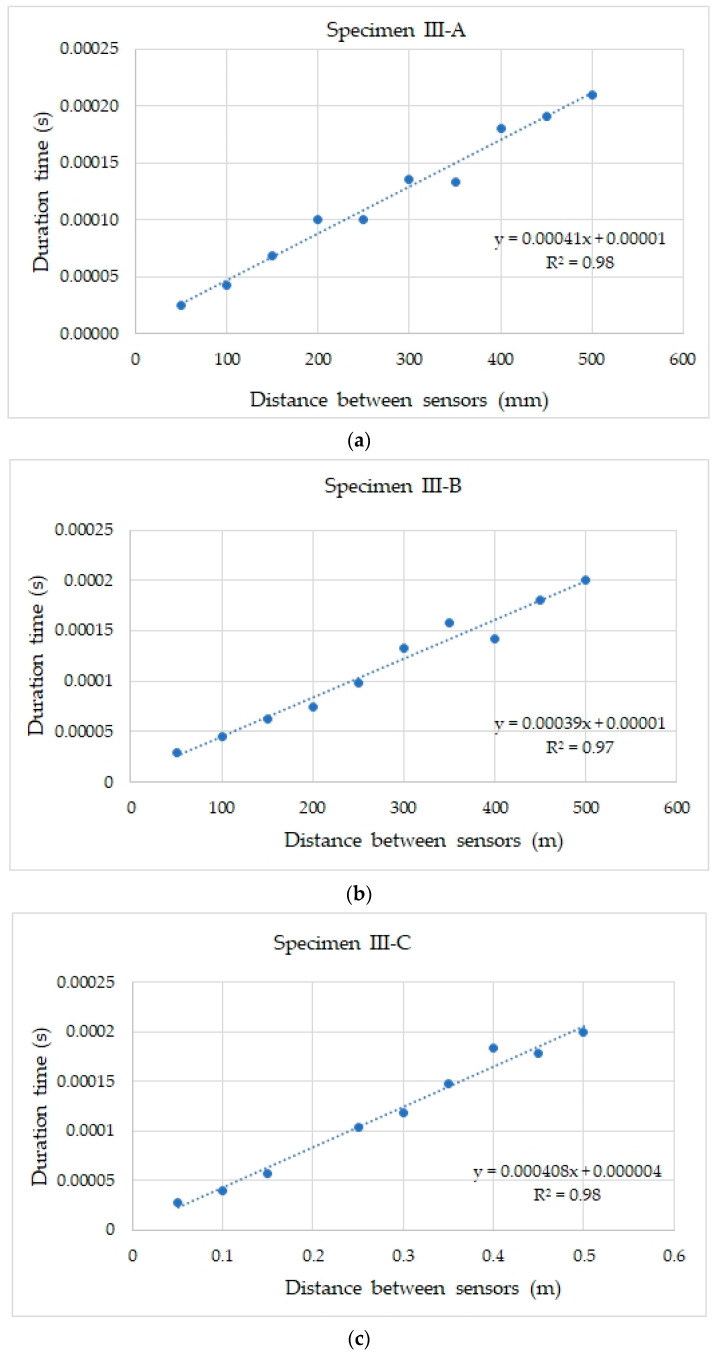
Dependency of duration vs. distance between sensors in specimens: (**a**) III-A, (**b**) III-B, and (**c**) III-C.

The acoustic wave velocity was estimated based on the guidelines of the standard [50] for testing concrete, using the ultrasonic method. For this purpose, for each sample, the relationship between the signal duration and the distance between the transmitting and receiving sensors is summarised and presented in Figure 15.

The results summarised in Figure 15 for each sample show exceptionally good agreement with the trend line, which was confirmed by the high coefficient of determination (above 0.97) in each case. Table 5 lists the acoustic wave velocities recorded in samples III-A, III-B, and III-C for the positioning of sensor No. 3 (Figure 9c), determined based on the curves developed according to [50], and are shown in Figure 15.

The estimated acoustic wave velocity values (Table 5) obtained during the indirect measurement for the positioning of sensor No. 3 (Figure 9c) were significantly lower than the velocity values listed in Table 3, which were determined directly for the positioning of sensor No. 1 (Figure 9a) and No. 2 (Figure 9b). This may be due to the different path over which the wave travels to reach the receiving sensor. A lower recorded acoustic wave velocity at the indirect position may indicate the receiving surface or transverse waves. These waves may be more sensitive to the influence of discontinuities in the surface zone.

**Figure 16:**
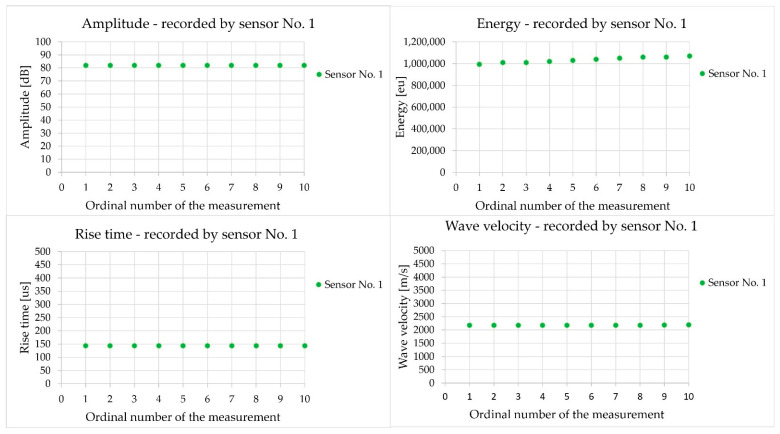
The results of amplitude, energy, rise time, and acoustic wave velocity generated by the AST function during ten consecutive measurements were recorded in specimen III-A, with the sensors positioned on the side wall of the sample at a distance of 150 mm.

Figure 16 shows the results of the acoustic wave parameters, that is, amplitude, energy, rise time, and wave velocity, recorded in sample III-A with the sensors placed on the sidewall at a distance of 150 mm.

The analysis of the parameters recorded in the indirect (Figure 16) and direct measurements (Figure 12b) with a sensor spacing of 150 mm showed the influence of sensor positioning on the parameters of the acoustic wave received. Comparing the same sensor distance in the axial arrangement and on the side surface, it can be observed that the amplitude remained unchanged at approximately 80 dB, the energy increased by 57%, the rise time increased by 18%, and the wave velocity decreased by 36%. Significantly higher energy values were recorded when the sensors were positioned on the same side of the surface (indirect measurement), which may indicate the influence of surface waves that propagate over the surface without energy loss. In the arrangement of the sensors facing each other (direct measurement), the energy is lower owing to greater losses in the material bulk caused by wave dispersion and attenuation. The longer rise time and lower wave velocity for the arrangement of the sensors positioned on the same side surface may be caused by the mixing of volumetric waves (longitudinal and transverse) with the Rayleigh surface wave, along with the associated effects of wave interference, reflection, and dispersion at heterogeneous sites within the material (aggregate grains, pores, and microcracks). The fact that a higher energy, which may be related to a smaller geometric loss over the surface, was observed indicates a strong contribution of the surface waves to indirect sensor positioning.

**Figure 17:**
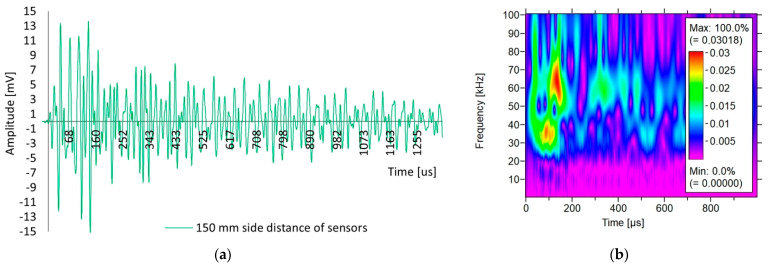
The results of: (**a**) the waveform analysis; (**b**) the wavelet analyses of the acoustic wave generated by the AST function in sample III-A with the sensors positioned on the side wall at a distance of 150 mm (indirect positioning).

Figure 17a shows the shape of the acoustic wave received from sample III-A by the sensor positioned on the sidewall at a distance of 150 mm from the other sensors.

Figure 17b shows the results of the wavelet analysis for sample III-A for an acoustic wave recorded by the sensors located on the side wall with 150 mm spacing. The results of the Wavelet Transform (WT) coefficient on 2D time-frequency maps received by one of the sensors are presented. The colours on the maps indicate the intensity of the wavelet transform coefficients of the sensor signal.

In Figure 17a, when the sensors were arranged on the side wall at a distance of 150 mm (indirect measurement), the rise time was 143 µs, and the amplitude was 15 mV. Compared to the axial positioning of the sensors (Figure 13b), with a distance of 150 mm separating the sensors, rise times of 121 µs and amplitudes of 11 mV were obtained. The difference in the value is due to the positioning of the sensors in relation to each other, which is also noticeable in the waveform. On the side wall, the waveform (Figure 17a) rises abruptly with peaks, with a long rise time and an amplitude value that is 36% higher, which is related to the higher energy compared to the axial positioning of the sensors. The surface and transverse waves likely propagated over the surface and maintained high frequency.

Based on Figure 17b, differences in the values of the WT coefficient can be observed for the waves received by the sensor, depending on the positioning of the sensors. In the case of direct positioning of the sensors (Figure 13d), a low coefficient of 0.022 was observed, whereas in the case of indirect positioning (Figure 17b), the coefficient was higher than 0.030, which gives an increase of 36%. This was related to the lower geometric loss on the surface of the sample. Based on this analysis, it appears that the lateral positioning of the sensors (indirect) results in a higher intensity. Energy is retained at the beginning of the wave, and visible ridges reach low frequencies of 25 kHz and high 80 kHz frequencies, with a dominant frequency of 65 kHz, which is likely caused by a larger proportion of surface waves than transverse waves. The axial arrangement of the sensors (Figure 13d) resulted in a lower intensity, and the energy also appeared at the beginning of the wave and was distributed over several ridges in the range of low 25 kHz and high 80 kHz frequencies, with a dominant frequency of 35 kHz, which is likely caused by a volumetric (e.g., longitudinal) wave.

### 3.2. Results of Hsu–Nielsen Tests

The analysis aimed to compare the parameters of the wave excited in the sample by breaking a pencil lead (Hsu–Nielsen source) at distances of 50, 100, 150, 200, and 250 mm from the nearest sensor (No. 1). Figure 10 shows a diagram of the positioning of the sensors and points at which an acoustic wave was excited in a given sample.

**Figure 18:**
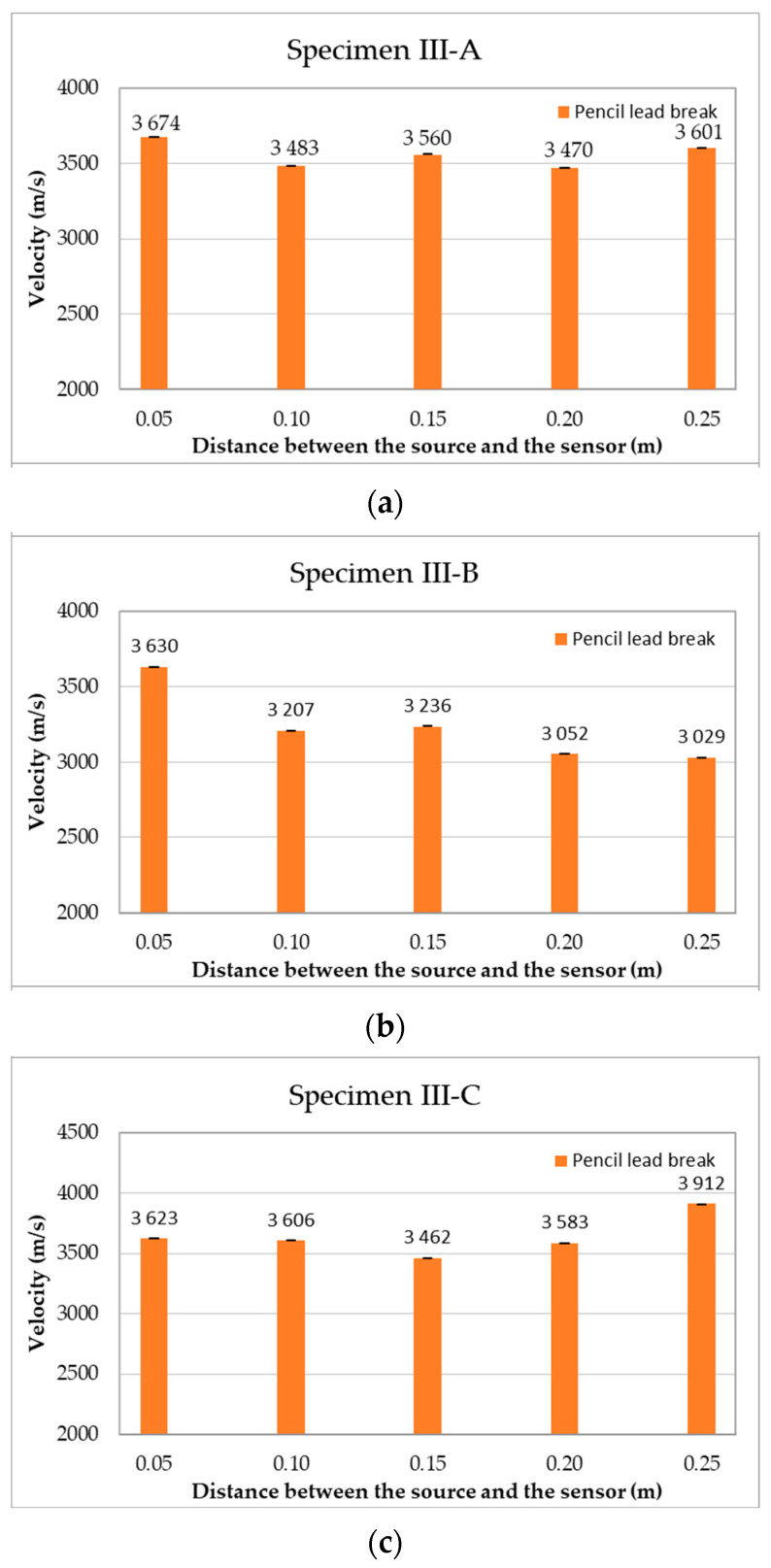
The mean velocity of the acoustic wave generated by the Hsu–Nielsen source depends on the distance between the signal source and the sensor in the samples: (**a**) III-A, (**b**) III-B, and (**c**) III-C.

The results of the acoustic wave velocity recorded as mean values (the mean value was calculated from the two sensors placed on the sample) at subsequent measurement points are shown in Figure 18.

To determine the variability of the results obtained, the mean velocities recorded in each of the samples at five measurement points were determined, along with the statistical parameters, and are compiled in Table 6.

The analysis showed an exceedingly small variation (3%) in the results for samples III-A and III-C. In the case of sample III-B, the coefficient of variation was 6.67%, which indicates a greater variability in the results and may be caused by inaccurate measurements.

**Figure 19:**
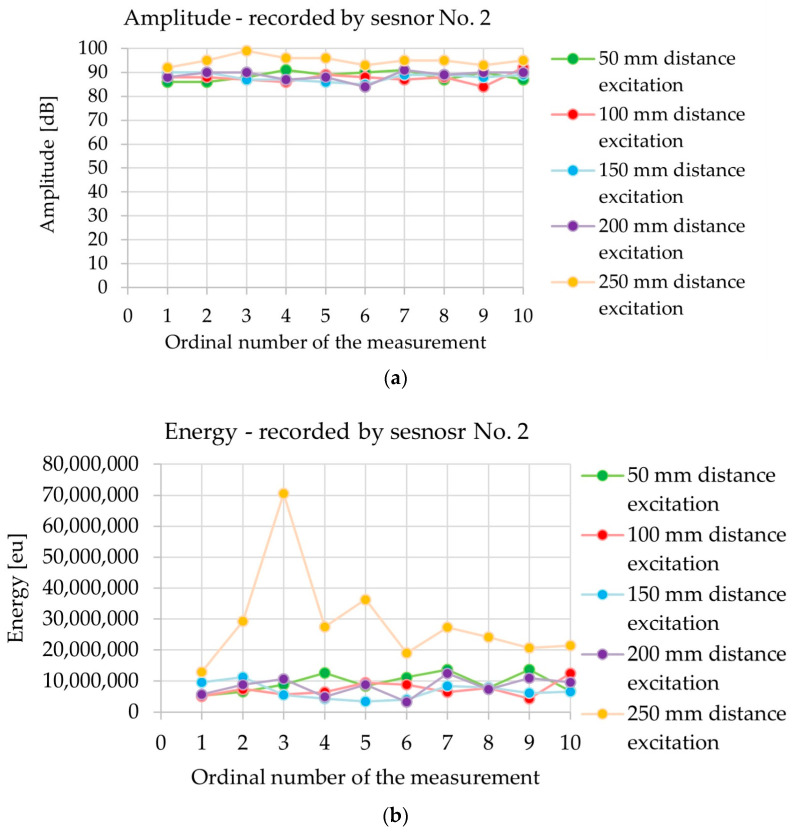

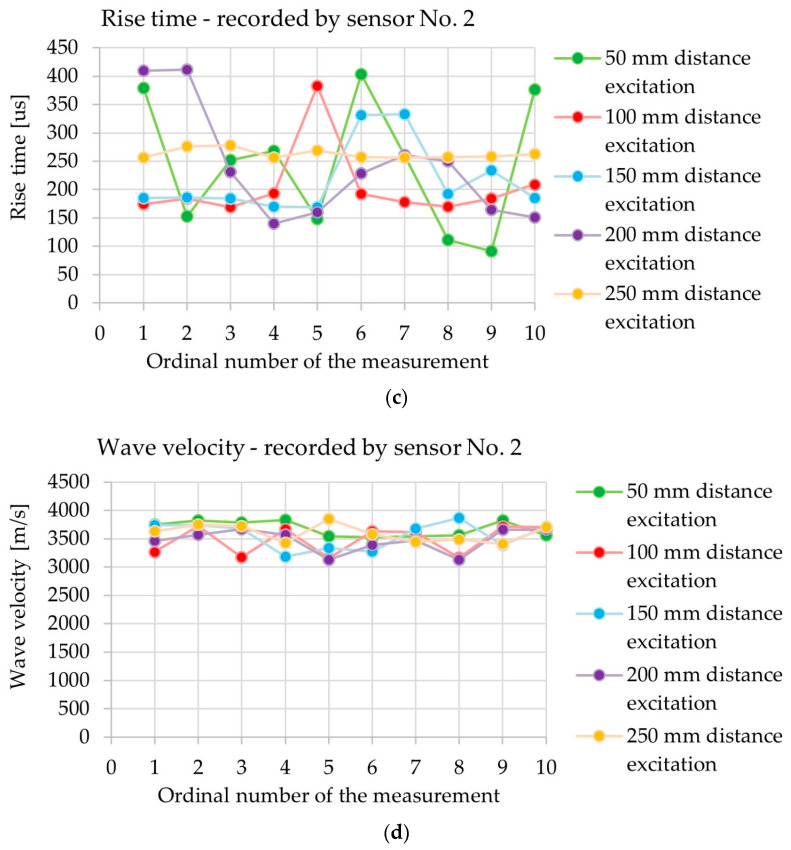
The results of the acoustic wave parameters generated by the Hsu–Nielsen source across 10 consecutive measurements in sample III-A, depending on the distance between the excitation source and sensor: (**a**) amplitude, (**b**) energy, (**c**) rise time, and (**d**) wave velocity.

Figure 19 shows a summary of the acoustic wave parameters: amplitude, signal energy, rise time, and velocity of the acoustic wave recorded during one measurement (10 consecutive fractures of pencil lead) in sample III-A at incrementally increasing distances from sensor No. 1 (Figure 10): 50, 100, 150, 200 and 250 mm.

In sample III-A, the amplitude (Figure 19a) was nearly constant, averaging 88–89 dB for wave excitation between 50 and 200 mm from sensor No. 1. It was only in the case of excitation at a distance of 250 mm from sensor No. 1 that the amplitude increased to 95 dB on average, which equates to a rise of 6 dB compared with the excitation at a distance of 50 mm. The energy (Figure 19b) increased along with the distance between the wave excitation point and the sensor No. 1: on average, 9.38 million eu (50 mm), 7.40 million eu (100 mm), 6.74 million eu (150 mm), 8.29 million eu (200 mm) and 28.90 million eu (250 mm). This represents an increase of +208% between 50 and 250 mm and +329% between 150 and 250 mm, with a single peak of 70.5 million eu for 250 mm. The energy dispersion rate for wave excitation at a distance of up to 200 mm is small and similar between the individual distances from sensor No. 1. At a distance of 250 mm from the point of excitation, the dispersion enters a sharp increase owing to a single event with extremely high energy. A potential reason for this result may be that the proximity of the edge of the sample is conducive to the interference and reflection of waves, differences in the location and strength of the pencil lead fracture, or a greater contribution from the surface wave. The rise time (Figure 19c) slightly increased with the distance between the wave excitation point and sensor No. 1 in the range of 100–250 mm: on average, 204 µs (100 mm), 217 µs (150 mm), 241 µs (200 mm), and 263 µs (250 mm). An exception can be found in the excitation of the wave at 50 mm from sensor No. 1, for which the mean rise time of 244 µs was similar to that of the wave excitation at a distance of 200 mm. A high variability in the rise time was observed for wave excitation in the range of 50–200 mm from sensor No. 1, which may be influenced by the geometry of wave propagation, superimposition of volumetric and surface waves, susceptibility to the place and force of the pencil lead fracture, and the heterogeneity of the concrete. In the case of wave excitation at a distance of 250 mm, the spread of the rise time decreases and is the most repetitive, which confirms a more stable wave propagation path, for example, one across the surface for further excitation from the sensor, and a greater share of the surface wave. The wave velocity (Figure 19d) was almost constant at an average level of 3470–3674 m/s for wave excitation at a distance of 50–150 mm from sensor No. 1. The mean wave velocity was 3558 m/s, which indicates the dominance of the longitudinal wave and not the transverse wave. The differences in wave velocity remain within ±5%, which may result from the variable share of volumetric waves (longitudinal and transverse), their reflections and overlapping waves, or the heterogeneity of the concrete (pores, cracks, and humidity). Wave velocity is a property of the material medium, and the dependence of the excitation distance on sensor No. 1 is statistically insignificant.

The variation in the values obtained (Figure 19) may be significantly influenced by the inaccuracy of the measurement, for example, by minimal displacements of the place of the pencil lead fracture, by the variable force of the pressure breaking the pencil lead, or by a different quality of the pencil lead. The impact of the manner in which the researcher handles the test station on the accuracy of the results obtained was emphasised in [51], and in [52], it was mentioned that even damage or wear of the Teflon pencil tube may affect the results obtained.

**Figure 20:**
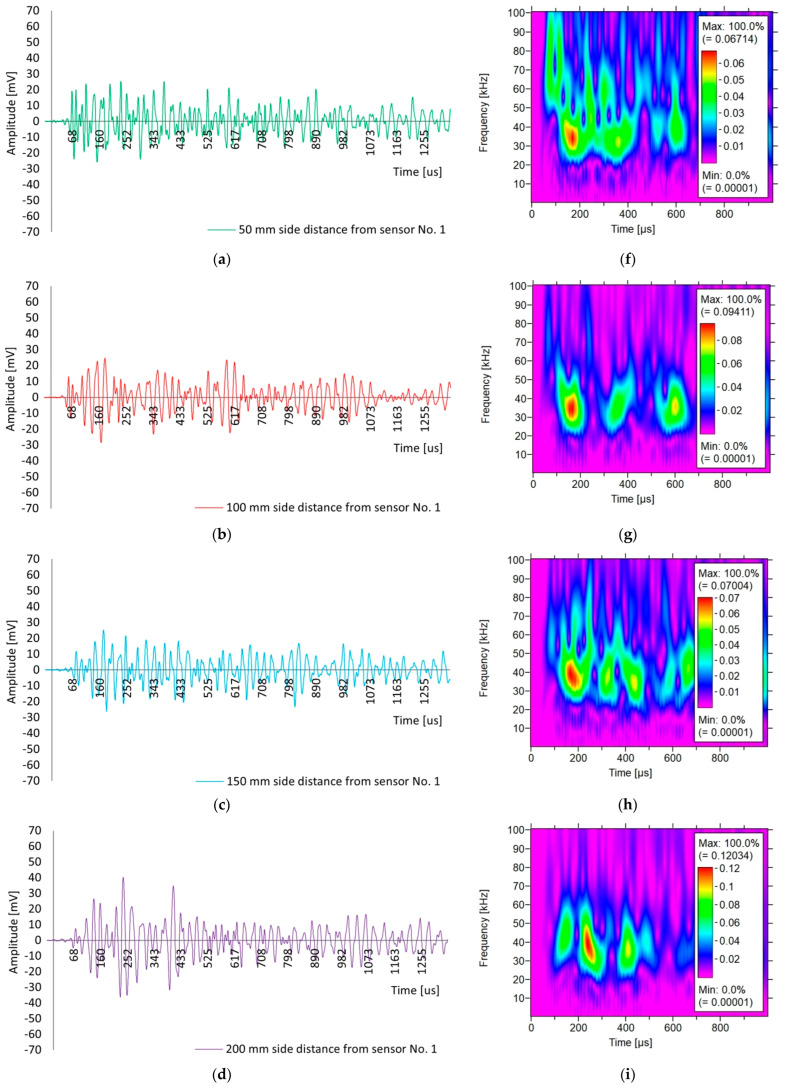

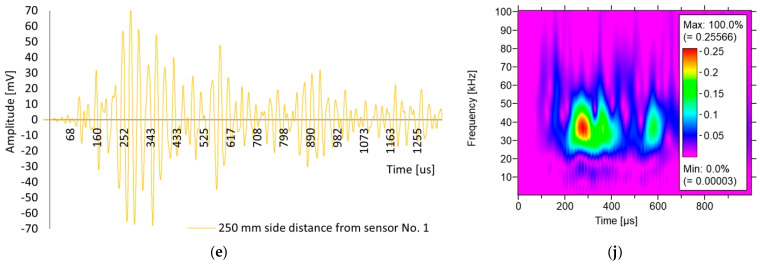
The results of waveform analysis (on left) applied to the acoustic wave generated in sample III-A by the Hsu–Nielsen source at a distance from the sensor No. 1: (**a**) 50 mm; (**b**) 100 mm; (**c**) 150 mm; (**d**) 200 mm; (**e**) 250 mm, and the results of the wavelet analysis (on right) applied to the acoustic wave generated in sample III-A by the Hsu–Nielsen source at a distance from the sensor No. 1: (**f**) 50 mm; (**g**) 100 mm; (**h**) 150 mm; (**i**) 200 mm; (**j**) 250 mm.

Figure 20 shows the shapes of the recorded waves generated by the Hsu–Nielsen test in sample III-A at incrementally increasing distances from sensor No. 1 (Figure 10): 50, 100, 150, 200 and 250 mm. It should be noted that the selection of the waveform was subjective because of the significant differences in the scatter of the data from the measurement of the wave surge time (Figure 19c).

Based on Figure 20, differences can be observed in the shape of the waves received by sensor No. 2, depending on the excitation distance from sensor No. 1. Increasing the excitation distance from sensor No. 1 from 50 to 250 mm changed the waveform and its parameters, that is, the peak amplitude increased from 25 to 70 mV (an increase of 180%), and the rise time increased from 153 to 276 µs (an increase of 80%). Short excitation distances (50–150 mm) yield short and sharp wave pulses with lower amplitudes of 15–28 mV. Long excitation distances (200–250 mm) give high and long pulses with larger amplitudes of 40–70 mV and high energy (Figure 19b). The farther away from the excitation source (200–250 mm), the greater part of the energy reaches the surface of the sample as a surface wave. A wave likely runs across the surface on which the sensor is located and aggregates other waves reflected from the edges and corners, as well as their energy. This increases the peak amplitude, energy, and rise time of the wave. At short excitation distances (50–150 mm), the influence of the source itself and local conditions, for example, the place and force of the pencil lead fracture, as well as concrete heterogeneity (pores, cracks, humidity), probably dominates.

Figure 20 also shows the results of the wavelet analysis for the selected sample III-A for an acoustic wave recorded by sensor No. 2 when Hsu–Nielsen excited the source at a distance from sensor No. 1: 50 mm (Figure 20f), 100 mm (Figure 20g), 150 mm (Figure 20h), 200 mm (Figure 20i), and 250 mm (Figure 20j). The results of the Wavelet Transform (WT) coefficient on 2D time-frequency maps received by one of the sensors are presented. The colours on the maps indicate the intensity of the wavelet transform coefficients of the sensor signal.

Based on Figure 20f–j, differences in the values of WT can be observed for the waves received by sensor No. 2, depending on the excitation distance from sensor No. 1, that is, 50, 100, 150, 200, and 250 mm. The maximum value of the WT coefficient tended to increase with the excitation distance, that is, 0.067 (50 mm), 0.094 (100 mm), 0.070 (150 mm), 0.120 (200 mm), and 0.256 (250 mm). The value of the wave coefficient shifted from 40% to 280% compared with the first measurement. In all cases, the frequency in the range of 25–50 kHz dominates, with excitation distances of 50 mm (Figure 20f) and 150 mm (Figure 20h). There appears to be more dissipated energy above 60 kHz, which disappears rapidly. In the case of excitation at a distance of 250 mm (Figure 20j), the energy was concentrated in a continuous and high ridge, which lasts for 200–400 µs. The change in the WT wave coefficient is consistent with an increase in energy (Figure 19b) and rise time (Figure 19c), and with a surge in the amplitude of the peak wave (Figure 20e) in the case of wave excitation at a distance of 250 mm from sensor No. 1. A longer excitation distance from sensor No. 1 increased the share of the surface wave received by sensor No. 2 on the surface and aggregated the reflections of waves from the edges through their superimposition.

### 3.3. Results of the Theoretical Values of Velocity

To determine the type of wave received, the theoretical values of the waves calculated according to formulas 1, 2, and 3 were determined based on [13], adopting the values of density (2540 kg/m^3^) and Young’s modulus (30 GPa) obtained experimentally as well as considering three Poisson’s coefficients, characteristic of concrete: 0.15, 0.20, and 0.25.

Longitudinal wave velocity:

(1) $$V_{L}=\sqrt{\frac{E\left(1-\nu \right)}{(1+\nu )\left(1-\nu \right)}}.$$

Shear wave velocity:

(2) $$V_{S}=\sqrt{\frac{E}{2\rho (1+\nu )}}.$$

Rayleigh wave velocity:

(3) $$V_{R}\approx \sqrt{\frac{E}{2\left(1+\nu \right)\rho }}\cdot \left(\frac{0.87+1.12\nu }{1+\nu }\right).$$

**Figure 21:**
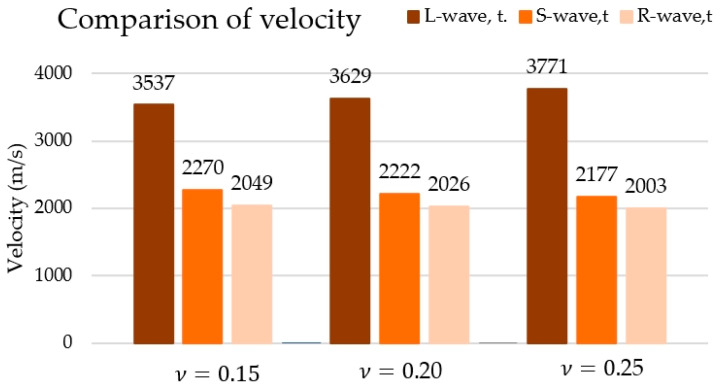
The theoretical longitudinal and transverse wave velocities of Rayleigh depend on the adopted Poisson’s coefficient.

The velocity values calculated based on Poisson’s coefficient are summarised in Figure 21.

Based on the results shown in Figure 21, it can be concluded that along with the value of the adopted Poisson’s coefficient, the velocities of the longitudinal wave (L-wave) increase, and those of the transverse waves (S-wave) and Rayleigh (R-wave) decrease.

The velocity values recorded using the AST method in direct measurement (Table 3) and using the Hsu–Nielsen method (Table 6) were similar to the velocity values of longitudinal waves calculated according to formula (1). In the case of velocities obtained using the AST method, during the indirect measurement, significantly lower velocity values were obtained (Table 5), which are closer to the transverse and surface wave velocity values calculated according to formulas (2) and (3) and are summarised in Figure 21.

## 4. Discussion

Analysis of the research results provided information on the possibility of using the AST function built into the PK6I sensors commonly used in the diagnostics of the structure to excite an acoustic wave to analyse its parameters, such as velocity, amplitude, rise time, and energy, as well as the analysis of waveforms and waveform intensity maps as an alternative artificial source.

### 4.1. Discussion of the Research Findings

The results of the measurements performed on the wave emitted from the sensor equipped with the AST function were characterised by high repeatability. A coefficient of variation below 1% when determining parameters such as velocity, amplitude, energy, or rise time denotes low variability of the results; therefore, their reliability is significant both with the arrangement of direct sensors (sensors set along an axis) and indirect sensors (sensors positioned on one wall).

In this study, the impact of positioning the sensors (direct/indirect) on the test element, influencing the type of recorded acoustic wave excited by an artificial source in the form of an auto-sensor test, was observed. In the case of positioning the sensors directly opposite each other so that the mass centres of the sensors were on one axis, either at a measurement distance of 150 or 600 mm, similar velocity values were obtained, differing by a maximum of 2.3% (Table 3). This indicates both the repeatability of the measurement method and the high homogeneity of the concrete. Reducing the sensor spacing from 600 mm to 150 mm caused a shift in the wave parameters; that is, the energy increased by 136% and the rise time decreased by 70% (Figure 12). The shorter propagation distance of 150 mm resulted in less wave attenuation and less impact of the reflected and overlapping waves. The waveform changes and with a sensor spacing distance of 600 mm, the wave rise time was longer than 403 µs, and the peak amplitude was low at 7 mV (Figure 13a); for the 150 mm spacing, the waveform was sharp, the rise time was shorter at 121 µs, and the peak amplitude was high at 11 mV, while maintaining high-frequency components up to 80 kHz (Figure 13b). Wavelet analysis showed that the wavelet coefficient WT increased by 46% from 0.015 to 0.022 when the sensor spacing was reduced. For a spacing distance of 150 mm, the energy appeared at the beginning of the wave and was concentrated in the 30–70 kHz band, in contrast to the 600 mm spacing, where the ridges were lower and shifted towards the frequency of 35 kHz (Figure 13). To determine the wave velocity, both sensor spacings of 150 mm and 600 mm are equivalent, while for the quality of the waveform, a spacing of 150 mm is preferable, considering a clear beginning of the wave, a short rise time, and an early onset of energy.

Significant differences were observed between the velocity values obtained using direct and indirect methods. At a distance of 150 mm between the sensors, the ratios of the velocity determined using the indirect method (position No. 3, Figure 9c) and direct (position No. 2, Figure 9b) were 0.65, 0.7, and 0.8, respectively, in samples III-A, III-B, and III-C. Similar differences were observed in concrete tests using the ultrasonic method. For example, in [53], the impact of the distance between the transducers on the wave velocity was studied, and it was found that the ratio of the velocity in the indirect method to the direct method reaches a value of 0.87 at a distance of 200 mm. However, ref. [54] shows that the differences in results between the direct and indirect methods can be up to 40%, which makes the indirect method less useful in some cases. These differences are due to the nature of the waves propagating in the concrete and their diffraction and interference, depending on the positions of the transmitting and receiving sensors. In the direct (axial) position of the sensors, the wave generated by the transmitter using the AST function travels in a straight line to the sensor receiver, travelling the shortest path in the inner zone, where the concrete is more homogeneous, does not dry out quickly, and where cement hydration occurs to a greater extent owing to the persistent moisture. The longitudinal wave (L-wave), which is characterised by the highest velocity and vibrations along the direction of propagation, is the most important in this case. In contrast, transverse waves (S-waves) are characterised by a lower velocity and direction of vibration of particles perpendicular to the direction of motion [55]. This type of wave can be dominant with the position of the sensors on one wall, when the wave is emitted deep into the concrete, towards the edge from which it bounces, in accordance with Huygens’ principle, which was analysed in [56]. Reflected waves may overlap and interfere with reading. The path of the wave from the transmitting to the receiving sensor, with the sensors placed on one wall, was longer than the distance between the sensors [55]. In addition, the propagation of surface waves occurs in a near-surface zone with greater porosity, sometimes with microcracks resulting from desiccation. Therefore, attenuation, diffraction, and reflection of waves occur at the surface, and slower surface waves are dominant. The other parameters of the elastic wave also differed under the same conditions. In the indirect positioning of the sensors, the energy was higher by 57% (Figure 16), the rise time was longer by 18% (Figure 16), and the wave velocity was reduced in relation to the direct positioning of the sensors (Figure 12b). The analysis of the waveform for the indirect positioning of the sensors showed clear changes in the steep and abrupt beginning of the wave, characteristic of transverse and surface waves, and a peak amplitude higher by 36% (Figure 17a) compared to the direct positioning of the sensors (Figure 13b). Wavelet analysis in the case of the indirect positioning of the sensors (Figure 17b) showed that the WT coefficient was 36% higher than that of the direct positioning of the sensors (Figure 13d). The visible ridges (Figure 17b) last longer at a frequency of 65 kHz, carrying most of the energy, which is likely caused by a larger proportion of surface waves and a smaller proportion of transverse waves in the recorded shape (Figure 17a). The indirect method is sensitive to the path of wave propagation at the surface owing to reflection, wave refraction, or proximity of the edge; therefore, the authors of the study recommend a direct method to precisely determine wave velocity.

**Figure 22:**
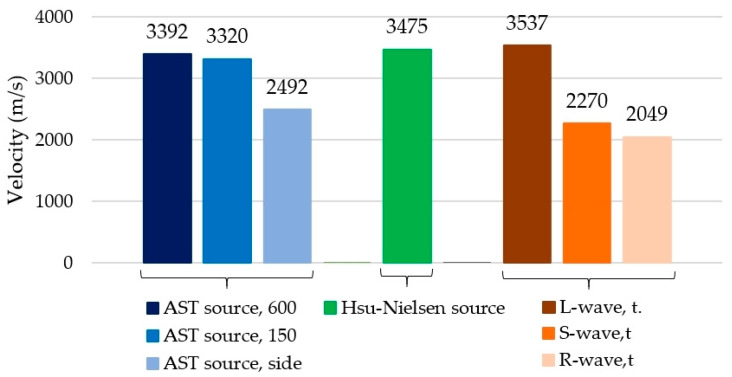
Comparison of the mean acoustic wave velocity excited by the AST function and the Hsu–Nielsen source from three samples (III-A, III-B, and III-C).

The values of the acoustic wave velocity generated in concrete using the AST method were compared with the values obtained using the Hsu–Nielsen method and theoretical velocity values. Figure 22 shows the mean velocity values determined from three concrete samples, III-A, III-B, and III-C, depending on the wave generation source (AST source or Hsu–Nielsen source). The results marked in the graph as “AST source—600” refer to the velocity obtained during direct measurement with the sensors positioned at a distance of 600 mm from one another, in accordance with the diagram in Figure 9a. The designation “AST source—150” refers to the measurement made when the sensors are positioned opposite to each other at a distance of 150 mm, in accordance with the diagram shown in Figure 9b. “AST source—side” denotes the velocity estimated according to the approach of the standard [50] on the basis of the indirect measurement performed with the sensors set on the side wall according to the diagram shown in Figure 9c. “L-wave, t.”, “S-wave t. “, and “R-wave t. “, were determined by Rayleigh’s longitudinal, transverse and surface velocities, respectively, determined based on formulas (1–3) adopting a Poisson’s coefficient value of 0.15. By assuming that Poisson’s coefficient is 0.15 in the calculations, the best match of the experimental values (both those obtained with the Hsu–Nielsen method and using sensors with the AST function) with the theoretical values was achieved (Figure 21). The variations in the case of longitudinal wave velocity were 4% when excited by the sensor, 2% when excited with the Hsu–Nielsen method, and, in the case of transverse wave velocity, 1% when excited by the sensor. In the case of higher values of Poisson’s coefficient, larger differences between the velocity values were obtained.

Based on the values obtained (Figure 22), it can be concluded that the wave velocities generated in concrete by PK6I sensors with the AST function, when directly measured with the sensors facing each other, are closest to the wave velocity generated with a pencil (Hsu–Nielsen source) and the theoretically determined velocity of the longitudinal wave (L-wave). In the case of the velocity of the waves generated in concrete by the PK6I sensors (with the AST function) positioned on the side wall of the sample (not axially relative to each other), the velocity obtained was much lower and closer to the theoretical values of the transverse or surface waves. When the wave was excited by the Hsu–Nielsen source with sensors positioned on the same wall (300 mm spacing), the increase in the excitation distance from 50 to 250 mm did not statistically influence the mean velocity. It is more likely that the observed variations of ±5% of single wave velocity measurements (Figure 19d) result from the overlapping and reflection of propagating waves through the interior of the concrete and on the surface, rather than from a change in the properties of the material. In this arrangement, the wave velocity is virtually independent of the distance between the wave source and sensor. Simultaneously, the wave parameters with an upward trend, such as energy (Figure 19b), rise time (Figure 19c), peak amplitude (Figure 20a–e), and wavelet coefficient WT (Figure 20f–j), change. This means that when the excitation occurs further from the sensor (50–250 mm), a part of the wave travels longer across the surface, amplifying the surface wave, which carries more energy. In addition, further excitation of the wave improves its quality, that is, a clear waveform, which translates into easier recording and analysis.

### 4.2. Advantages and Limitations of Testing Using AST

The results presented in this work confirm the legitimacy of applying the Auto Sensor Test (AST) function in PK6I sensors as an alternative signal source in studies of acoustic emission in concrete. The obtained data demonstrated high repeatability and compliance with the reference values of the wave velocity obtained using the Hsu–Nielsen test. Originally, the Manufacturer envisaged the use of an integrated AST function in PK6I sensors to simulate acoustic emission waves as a means of evaluating the performance of the sensors and their connection to the evaluated material. This paper presents research that extends the use of sensors with AST functions. The possibility of using a simulated wave of acoustic emission sent from the sensor for the purposes of the measurement and analysis of wave velocity changes in a material and wave parameters (i.e., energy, rise time, amplitude, waveform analysis, and wavelet analysis) was confirmed. These tests are important as they are the basis for in situ diagnostics of concrete, including the detection of damage and the assessment of concrete homogeneity. They have the potential to improve non-destructive testing of concrete and other materials, such as sustainable construction materials. The integrated AST function in the sensor is a practical and quick tool for performing tests, owing to the repeatability and low variability of the results. Wave excitation occurs remotely without the operator’s interference in the medium, eliminating human error, allowing data to be compared between different facilities. Furthermore, the method does not incur costs with repeated use, and electronic control reduces signal source variability because the transducers can be permanently mounted on the tested element. The acoustic emission measurement system presented in this work is particularly useful in field conditions and in locations with limited access, owing to the portability of the device, that is, small dimensions, low weight, and ease of transport.

Despite the numerous advantages and possibility of using it without incurring additional lost costs, the method of exciting an acoustic wave in concrete through the use of sensors with the AST function has its limitations, especially with the indirect arrangement of the sensors (on one wall). Their knowledge is crucial for the correct interpretation of results and the evaluation of measurement reliability.

For example, an excessively small distance between the sensors may reduce the value of the wave velocity, which may result from the non-stabilisation of the longitudinal wave form in the near-field zone, the impact of which on the velocity of an ultrasonic wave was discussed in the work [57] on the example of steel elements. In [58], it was demonstrated that the positioning of sensors in relation to the signal source affects the underestimation of ultrasonic wave velocity in tests conducted using the impact-echo method. In the case of ultrasonic methods, standard guidelines [50] indicate setting the minimum distance between the sensors and the signal source to 100 mm for more reliable results. The authors of the study also observed an underestimation of the received acoustic wave velocity during indirect measurements when the sensors were positioned on one wall with 50 mm spacing. Therefore, the minimum distance between the sensors recommended by the authors is 100 mm, which is confirmed by the guidelines in the standard [50] developed for the ultrasonic method. In addition, during indirect measurements (with the sensors placed on the side wall), a level of variability in the recorded velocity was observed, up to 8% at the measurement points, with distances between sensors ranging from 100 to 500 mm, even though the coefficients of variation between ten measurements made at a single point did not exceed 1%. The result of the recorded acoustic wave velocity with the sensors positioned on one sidewall of the sample may be affected by the delay of the equipment, the large impact of which on the duration of wave passage in the case of, for example, the ultrasonic method, was discussed in [59]. For this reason, to determine the acoustic wave velocity when the sensors are positioned indirectly (on one wall), the authors of this study recommend the guidelines developed for the ultrasonic method and included in the standard [50], which involve taking several measurements on the sample at different distances and comparing the distance between the sensors with the signal duration. In such cases, the acoustic wave velocity was estimated based on the slope of the resulting curve.

### 4.3. Proposal for Further Research

Suggestions for further tests of concrete utilising sensors with the AST function:

- Analysis of test results for elements with varying degrees of degradation and cracking;
- Analysis of the impact of temperature and humidity changes, concrete age, type of cement, aggregate and the presence of reinforcement on wave propagation;
- Analysis of the impact of maximum sensor spacing on the results obtained;
- Analysis of the impact of sensors with varying resonance frequencies on the results.

It was demonstrated that, on account of the effectiveness and repeatability of acoustic wave parameter measurements in concrete, the method of wave induction by the sensor with the AST function could serve as an effective alternative to the Hsu–Nielsen method.

## 5. Conclusions

Based on the tests and analyses conducted, the following conclusions were drawn.

1. PK6I sensors with the AST function are capable of emitting and receiving an acoustic wave propagating in concrete, while the AST function can be used not only to calibrate the sensors and check their connection with concrete, but can also be employed in the analysis of the material under study.
2. The positioning of the sensors (on the side wall or opposite to each other) on the sample affects the type of acoustic wave recorded.
3. The direct positioning of the sensors (opposite to each other) during the test using a sensor with the AST function allows for recording a longitudinal wave in concrete, with velocity values similar to those obtained in the Hsu–Nielsen test and those estimated theoretically.
4. The indirect positioning of the sensors (on one wall) during the test using a sensor with the AST function allows for the recording of a wave travelling in concrete with velocities similar to those of transverse and surface waves estimated theoretically.
5. The wave velocity when positioning sensors with the AST function on one wall (indirect measurement) can be estimated according to the approach for the ultrasonic method proposed in [50].
6. The coefficient of variation of the wave velocity results obtained during 10-fold induction with a sensor equipped with the AST function was up to 1%, which was lower than the coefficient of variation obtained in the Hsu–Nielsen test at the same point (5.5%).
7. When performing direct measurements using the AST function (sensors positioned facing one another), a slight displacement of the sensors in relation to their axes does not significantly affect the obtained wave velocity results within a radius of 100–150 mm, that is, a shift between the mass centres of the sensors within a radius of up to 100 mm caused differences in velocity values of 4.3% and within 150 mm by approximately 6%.
8. The possibility of using changes in the selected parameters of the elastic wave emitted from a sensor with the AST function as an indicator of the damage and homogeneity of the concrete. The grounds for this conclusion stem from the fact that the energy, rise time, peak amplitude, and WT wavelet coefficient react to changes in path length and the type of wave propagation.
9. The test results confirmed that PK6I sensors with the AST function can serve as an alternative signal source for estimating the velocity of a propagating wave in concrete and analysing other parameters of the wave received, that is, the peak amplitude, rise time, and energy, as well as waveform analysis and wavelet analysis.

## Figures and Tables

**Table 1:** Category or declared values for selected parameters of basalt aggregate.

Parameter	Basalt Aggregate 2/8 [43]	Basalt Aggregate 8/16 [44]
Specific gravity (Mg/m^3^)	3.18	3.17
Water absorption (%)	0.9	0.7
Dust content (%)	1.5	1.5
Resistance to crushing	LA_15_	LA_15_
Resistance to abrasion	M_DE_10	M_DE_20
Resistance to polishing	PSV_50_	PSV_50_
Resistance to surface abrasion	AAV_10_	AAV_10_
Frost resistance	F_1_	F_1_
Reactivity of alkaline	Non-reactive	Non-reactive

**Table 2:** Performance properties and components of CEM III/A 42.5N-LH/HSR/NA blast furnace cement.

**Components and Properties**	**Values [45]**
Portland cement clinker (K)	35–50%
Granulated blast furnace slag (S)	50–65%
Secondary components	0–5%
Compressive strength class	42.5 N
Compressive strength after 2 days	Above or equal to 10 MPa
Compressive strength after 28 days	Between 42.5 MPa and 62.5 MPa

**Table 3:** The values of the acoustic wave velocity were measured using the direct method in two perpendicular directions, along with the differences between the values and statistical parameters in percentage points.

Number of Specimens	Mean Velocity (m/s) Measured from a Distance of 600 mm	Mean Velocity (m/s) Measured from a Distance of 150 mm	Difference in Velocity Values (%)
III-A	3425	3381	0.86
III-B	3361	3310	1.02
III-C	3383	3268	2.28
Mean value	3390	3320	1.38
Standard deviation	32	51	
Variation (%)	0.95	1.52	

**Table 4:** Statistical parameters of velocity results (m/s) for the tested specimens.

Statistical Parameters	Specimen III-A		Specimen III-B		Specimen III-C	
	Sensor No.1	Sensor No.2	Sensor No.1	Sensor No.2	Sensor No.1	Sensor No.2
Mean value (m/s)	3350	3388	3268	3243	3184	3178
Standard deviation (m/s)	86	25	99	83	84	81
Coefficient of variation (%)	2.6	0.7	3.0	2.6	2.6	2.6

**Table 5:** The acoustic wave velocities recorded in samples III-A, III-B, and III-C for positioning sensor No. 3 were determined according to [50].

Velocity (m/s)	Specimen III-A	Specimen III-B	Specimen III-C
Velocity calculated according to PN-EN 12504-4:2021-12 [50]	2433	2591	2451

**Table 6:** Mean values of wave velocity generated in concrete specimens by the Hsu–Nielsen source (mean value from five points) and statistical parameters of the results.

Statistical Parameters	Specimen III-A	Specimen III-B	Specimen III-C
Mean value of velocity (m/s)	3558	3231	3637
Standard deviation (m/s)	76	215	149
Coefficient of variation (%)	1.97%	6.67%	2.39%

## Data Availability

The original contributions presented in this study are included in the article. Further inquiries can be directed to the corresponding authors.
